# Does a nurse-led interventional program improve clinical outcomes in patients with atrial fibrillation? A meta-analysis

**DOI:** 10.1186/s12872-024-03707-3

**Published:** 2024-01-11

**Authors:** Xingcai Yu, Jun Xu, Min Lei

**Affiliations:** 1 Cardiac catheterization unit, Yantaishan Hospital, Yantai, Shandong 264000 People’s Republic of China; 2 Health Management Centre, Yantai Qishan Hospital, Yantai, Shandong 264001 People’s Republic of China; 3 Department of Nursing, Shaanxi Rehabilitation Hospital, Xian, Shaanxi 710065 People’s Republic of China

**Keywords:** Nurse-led clinics, Atrial fibrillation, Nurse-led intervention, Usual care, Cardiac outcomes, Stroke

## Abstract

**Background:**

Being the most common type of arrhythmia, atrial fibrillation (AF) is progressively increasing with an annual rate of 5 million new cases. Recent guidelines highlight the importance of using collaborative multidisciplinary teams in order to improve outcomes during management of patients with AF. A nurse-led program including a nurse-directed education, counselling and intervention has shown to improve patients’ outcomes in candidates with AF. In this analysis, we aimed to systematically compare the clinical outcomes observed in patients with AF who were assigned to a nurse-led interventional program versus a usual care group.

**Methods:**

EMBASE, MEDLINE, Http://www.ClinicalTrials.gov, Web of Science; Google Scholar and Cochrane databases were the data sources. The clinical outcomes were considered as the endpoints in this study. This is a meta-analysis, and the statistical analysis was conducted by the RevMan software (version 5.4). Risk ratios (RR) with 95% confidence intervals (CI) were used to represent the data after statistical analysis.

**Results:**

Six studies with a total number 2916 participants were included whereby 1434 participants were assigned to a nurse-led intervention and 1482 participants were assigned to the usual care group. Our results showed that participants with AF who were assigned to the nurse-led interventional group had a significantly lower risk of composite endpoints (RR: 0.82, 95% CI: 0.70–0.96; *P* = 0.01), heart failure (RR: 0.66, 95% CI: 0.47–0.92; *P* = 0.02), atrial fibrillation (RR: 0.77, 95% CI: 0.63–0.94; *P* = 0.01) and re-admission (RR: 0.78, 95% CI: 0.62–0.99; *P* = 0.04). However, the risks of all-cause mortality (RR: 0.86, 95% CI: 0.68–1.09; *P* = 0.21), cardiac death (RR: 0.67, 95% CI: 0.33–1.39; *P* = 0.28), myocardial infarction (RR: 0.70, 95% CI: 0.35–1.42; *P* = 0.33), stroke (RR: 0.75, 95% CI: 0.44–1.26; *P* = 0.28), all bleeding events (RR: 1.11, 95% CI: 0.81–1.53; *P* = 0.51) and major bleeding events (RR: 0.91, 95% CI: 0.56–1.49; *P* = 0.71) were not significantly different.

**Conclusions:**

The nurse-led interventional program significantly improved composite endpoints including heart failure and the recurrence of AF, resulting in a significantly lower admission rate compared to the usual care group. However, nurse-led interventional program did not affect mortality, stroke, myocardial infarction and bleeding events. Based on our current results, a nurse-led interventional programs apparently could be beneficial in patients with AF. Future larger trials would be able to confirm this hypothesis.

## Introduction

Being the most common type of arrhythmia, atrial fibrillation (AF) is progressively increasing with an annual rate of 5 million new cases [1]. Complicated AF might result in stroke, heart failure and even death among patients [2]. Recurrent AF is common in patients with the disease, and consequently, AF is considered as a chronic condition. Accordingly, AF significantly increases healthcare burden and jeopardizes the quality of life of affected patients [3].

AF is considered to affect more than 33 million people globally, with 8.8 million in the European community, 3.9 million among the Chinese community and over 9 million in the United States [4]. The prevalence of AF is expected to double or even triple by 2030–2050 and this would represent a major healthcare challenge due to the association of AF with several life-threatening outcomes.

Most researches have selectively studied patients. For example, patients undergoing procedural interventions, AF related to surgery or pharmacological effect of certain drugs on AF outcomes were often studied. However, studies based on a more ‘general’ AF population and corresponding outcomes are scarce [5].

Recent guidelines highlight the importance of using collaborative multidisciplinary teams in order to improve outcomes during management of patients with AF [6–8]. A nurse-led interventional program including a nurse-directed education, counselling and intervention has shown to improve patients’ outcomes in candidates with AF [9]. However, the Nurse-led Atrial Fibrillation Management (NEAT) study showed that a brief nurse-delivered educational intervention did not significantly improve health related quality of life or risk factor status in patients with AF [10].

In this analysis, we aimed to systematically compare the clinical outcomes observed in patients with AF who were assigned to a nurse-led interventional program versus a usual care group.

## Methods

### Data sources

EMBASE, MEDLINE, Http://www.ClinicalTrials.gov, Web of Science; Google Scholar and the Cochrane databases were the data sources. Relevant publications were searched from those databases.

### Search strategies

The following search terms were used:

- ‘Nurse-led intervention and atrial fibrillation’;
- ‘Nurse-led clinic and atrial fibrillation’;
- ‘Nurse care and atrial fibrillation’;
- ‘Nurse clinics and atrial fibrillation’;
- ‘Nurse-led intervention versus usual care and atrial fibrillation’.

### Inclusion and exclusion criteria

Inclusion criteria were:

- Studies that compared nurse-led care versus usual care in patients with AF;
- Studies that reported cardiovascular outcomes as their clinical endpoints;
- Studies that were published in English.

Criteria for exclusion were:

- Studies that were systematic reviews, meta-analyses and literature reviews;
- Studies where a control group was absent for comparison;
- Studies that did not report cardiovascular outcomes;
- Studies that were not based on patients with AF;
- Studies that were published in a different language apart from English;
- Duplicated studies or studies that repeated themselves in different search databases.

### The endpoints which were assessed

The outcomes which were assessed included:

- Heart Failure;
- Cardiovascular mortality;
- All-cause mortality;
- Myocardial infarction;
- Stroke;
- Composite endpoint which was defined as a total number of events including heart failure, mortality, cardiac death, stroke, myocardial infarction and bleeding events;
- Recurrent atrial fibrillation;
- All bleeding events including any type of bleeding (minor + major bleedings);
- Major bleeding.

The endpoints which were reported in the original studies have been listed in Table 1.

**Table 1:** Outcomes which have been reported in the original studies

Studies	Outcomes	Type of AF	Regular Follow-up by nurse
**Caravaca2020 **[14]	Ischemic stroke/TIA, acute MI, mortality, major bleeding, clinically relevant non-major bleeding, any bleeding	Non-valvular AF	Follow up by nurse every 6 months
**Fuenzalida2017 **[15]	Heart failure, stroke or systemic embolism, anti-arrhythmic treatment-related complications, death, emergency visits, admissions	Mainly permanent AF followed by paroxysmal AF	3 months follow-up
**Hendriks2012 **[16]	Composite endpoints, cardiovascular deaths, cardiac arrhythmic, cardiac non-arrhythmic death, vascular non-cardiac death, cardiovascular hospitalization, arrhythmic events, atrial fibrillation, syncope, ventricular tachycardia, cardiac arrest, heart failure, acute MI, stroke, systemic embolism, major bleeding, life-threatening effects of drug	Mainly symptomatic AF followed by paroxysmal AF and then permanent AF	3, 6 and 12 months
**Inglis2004 **[17]	All-cause mortality, acute MI, angina, atrial fibrillation, congestive heart failure, stroke	Chronic AF	Regular follow-up during a period of 5 years
**Wijtvliet2020 **[18]	Composite endpoints, cardiovascular deaths, cardiac arrhythmic, cardiac non-arrhythmic death, vascular non-cardiac death, cardiovascular hospitalization, arrhythmic events, atrial fibrillation, syncope, ventricular tachycardia, cardiac arrest, heart failure, acute MI, stroke, systemic embolism, major bleeding, life-threatening effects of drug	First time detected AF, mostly paroxysmal AF	3, 6, 12 months and yearly follow-up
**Yan2022 **[19]	Cardiovascular hospitalization, atrial fibrillation, heart failure, stroke, ventricular tachycardia, cardiovascular death	Chronic AF	1, 3, 6, 12 months follow-up

*TIA* Transient ischemic attack, *MI* Myocardial infarction, *AF* Atrial fibrillation

Patients were followed up by the nursing team on a regular basis. The follow-up time periods have also been stated in Table 1.

### Data extraction and quality assessment

All the authors were involved in the data extraction process. They carefully studied the papers, and then independently extracted data from the original studies. First of all, the names of authors, publication year, the number of participants assigned to the nurse-intervention group and the usual care group respectively, the mean age of the participants, the mean percentage of male participants, the mean percentage of patients with diabetes mellitus, hypertension and current smokers were all extracted. In addition, the type of study (randomized or observational), the participants’ enrollment period, the mean percentage of participants using medications such as antiplatelet agents, calcium channel blockers, beta-blockers, angiotensin converting enzyme inhibitors, angiotensin-renin blockers, statins, diuretics and anti-arrhythmic drugs were all carefully extracted. The authors also extracted data reporting the clinical outcomes with the associated number of events.

Any disagreement which occurred during this data extraction process was carefully discussed among all the authors and a consensus was reached.

The quality assessment of the randomized studies was carried out based on the criteria suggested by the Cochrane risk of bias assessment [11] and the quality assessment of the observational cohort was carried out by the Newcastle Ottawa Scale (NOS) [12].

### Statistical analysis

This is a meta-analysis, and the statistical analysis was conducted by the RevMan software (version 5.4). Risk ratios (RR) with 95% confidence intervals (CI) were used to represent the data after statistical analysis.

Heterogeneity was assessed by the Q statistic test as well as the I^2^ statistical test. A subgroup analysis with a *P* value less than or equal to 0.05 was considered statistically significant whereas a result for a subgroup analysis having a *P* value greater than 0.05 was considered insignificant statistically. In addition, the higher the I^2^ value, the larger the heterogeneity.

A fixed effect statistical model was used for this analysis.

Sensitivity analysis was also carried out to ensure that the final result was not influenced by any particular study. Publication bias was assessed by visualizing funnel plots.

### Ethical approval

This is a meta-analysis including data from previously published studies. Therefore, an ethical or board review approval was not required.

## Results

### Search outcomes

The Preferred Reporting Items in Systematic reviews and Meta-analyses (PRISMA) guideline [13] was followed during this search process. Our search outcome resulted in a total number of 394 publications. The authors carefully assessed the titles and abstracts, and therefore, those articles which were not relevant to the scope of this research were directly eliminated. A total number of 82 full text articles were assessed for eligibility.

Based on the criteria for inclusion and exclusion, further eliminations were carried out. Studies were eliminated because they were:

- Systematic reviews or meta-analyses (2);
- A control group was absent (6);
- Even though the title reported nurse intervention in patients with AF, the comparison was not between a group with nurse intervention versus usual care (8);
- Respective endpoints were not reported. Instead, endpoints based on quality of life and treatment satisfactions were reported (8);
- Duplicated studies (52).

**Fig. 1:**
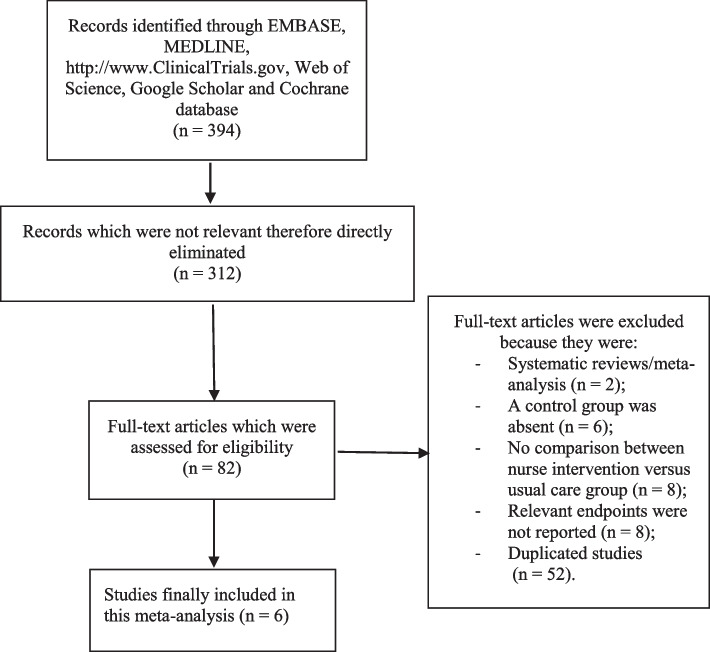
Flow diagram representing the study selection

Finally, a total number of 6 studies [14–19] were selected for this analysis. The flow diagram representing the study selection has been demonstrated in Fig. 1.

### General features of the studies

The general features of the studies have been listed in Table 2. A total number 2916 participants were included in this analysis, whereby 1434 participants were assigned to a nurse care intervention group and 1482 participants were assigned to the usual care group as shown in Table 2. The enrollment period was between years 2007 and 2020. Five studies were randomized trials and one study was an observational study.

**Table 2:** General features of the studies

Studies	Number of participants assigned to the nurse care (n)	Number of participants assigned to the usual care (n)	Type of study	Participants’ enrollment time period
**Caravaca2020**	107	116	Observational	2015–2017
**Fuenzalida2017**	116	124	Randomized	2011–2012
**Hendriks2012**	356	356	Randomized	2007–2008
**Inglis2004**	68	84	Randomized	–
**Wijtvliet2020**	671	683	Randomized	2012–2018
**Yan2022**	116	119	Randomized	2018–2020

### Baseline characteristics of the participants

The baseline characteristics of the participants have been given in Table 3. The mean percentage of male participants ranged from 42.2 to 67.0% with a mean age ranging from 64.0 years to 77.3 years. Co-morbid conditions and cardiovascular risk factors included diabetes mellitus (9.00–47.0%), hypertension (26.7–79.4%) and smoking history (27.8–41.1%) as shown in Table 3.

**Table 3:** Baseline features of the studies

Studies	Age (years)	Males (%)	DM (%)	HBP (%)	CS (%)
	NI/UC	NI/UC	NI/UC	NI/UC	NI/UC
**Caravaca2020**	72.5/76.5	54.7/54.3	38.3/47.0	79.4/79.1	41.1/27.8
**Fuenzalida2017**	74.8/77.3	42.2/42.7	18.1/25.0	68.1/67.7	–
**Hendriks2012**	66.0/67.0	55.3/62.1	14.0/12.9	52.5/54.2	–
**Inglis2004**	73.5/73.0	53.0/53.0	38.0/31.5	60.0/53.0	–
**Wijtvliet2020**	64.0/64.0	67.0/65.0	11.0/9.00	49.0/46.0	–
**Yan2022**	65.0/65.4	64.7/58.0	41.4/31.9	26.7/28.6	–

*DM* Diabetes mellitus, *HBP* High blood pressure, *CS* Current smoker, *NI* Nurse Intervention, *UC* Usual care

Medications which were used by the participants have been listed in Table 4. The mean percentage of participants who were taking anti-arrhythmic drugs ranged from 14.0 to 92.0%, and those who were on calcium channel blockers ranged from 5.10 to 24.1%, those on beta-blockers ranged from 15.1 to 77.6%, those who were on statins ranged from 27.8 to 59.8%, those taking diuretics ranged from 15.0 to 22.4%, those on angiotensin converting enzyme inhibitors/angiotensin-renin blockers ranged from 41.3 to 73.8%, and those taking antiplatelets ranged from 21.8 to 94.3% as shown in Table 4.

**Table 4:** Medication used by the participants

Medications	Caravaca2020	Fuenzalida2017	Hendriks2012	Inglis2004	Wijtvliet2020	Yan2022
	NI/UC	NI/UC	NI/UC	NI/UC	NI/UC	NI/UC
**Anti-arrhythmic**	14.0/14.7	85.78/84.94	58.1/41.9	92.0/90.0	–	69.0/73.1
**CCBs**	20.6/24.1	5.43/7.94	12.4/5.10	–	–	–
**BBs**	77.6/63.2	15.06/15.89	46.1/52.5	–	–	–
**Statins**	59.8/59.5	–	33.4/27.8	–	–	–
**Diuretics**	15.0/22.4	–	15.7/18.8	–	–	–
**Anti-platelets**	35.5/23.3	59.5/70.2	94.3/83.1	62.5/55.0	–	23.3/21.8
**ACEI/ARB**	73.8/70.7	–	44.9/41.3	–	–	–

*NI* Nurse Intervention, *UC* Usual care, *CCBs* Calcium channel blockers, *BBs* Beta blockers, *ACEI* Angiotensin converting enzyme inhibitors, *ARBs* Angiotensin receptor blockers

### Main results of this analysis

**Fig. 2:**
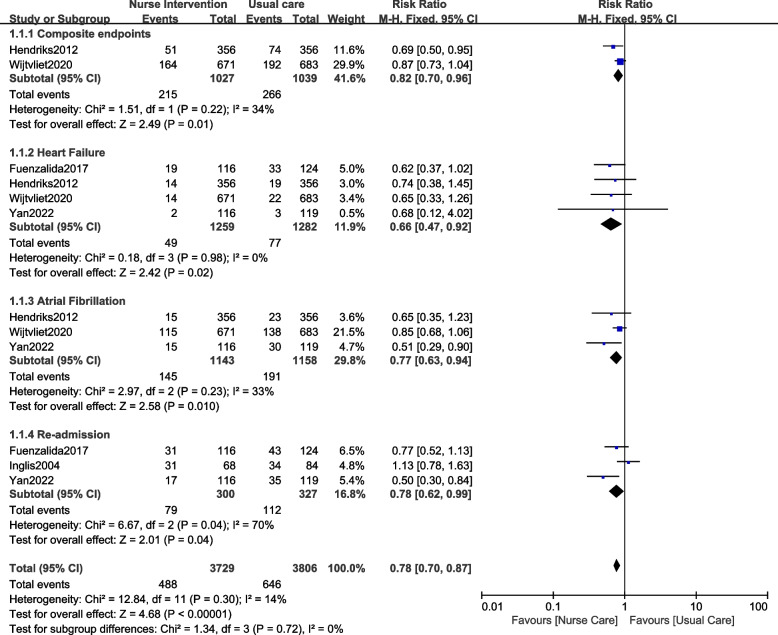
Clinical outcomes observed with nurse-led intervention versus usual care for patients with AF (A)

Our results showed that participants with AF who were assigned to the nurse-led interventional group had a significantly lower risk of composite endpoints (RR: 0.82, 95% CI: 0.70–0.96; *P* = 0.01), heart failure (RR: 0.66, 95% CI: 0.47–0.92; *P* = 0.02), atrial fibrillation (RR: 0.77, 95% CI: 0.63–0.94; *P* = 0.01) and re-admission (RR: 0.78, 95% CI: 0.62–0.99; *P* = 0.04) as shown in Fig. 2.

**Fig. 3:**
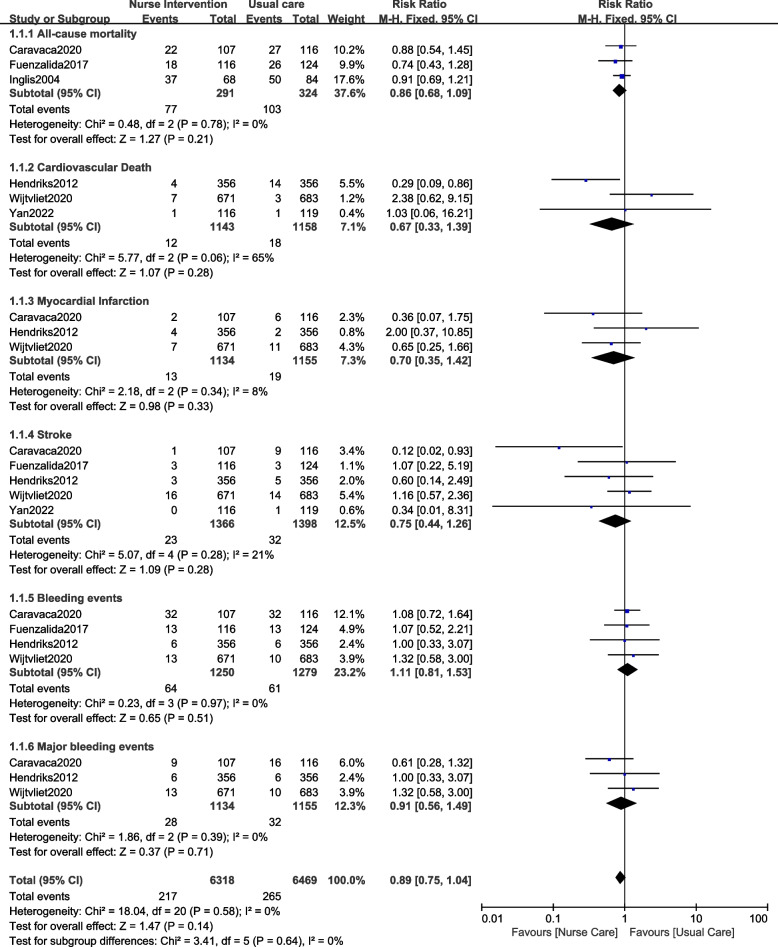
Clinical outcomes observed with nurse-led intervention versus usual care for patients with AF (B)

However, the risks of all-cause mortality (RR: 0.86, 95% CI: 0.68–1.09; *P* = 0.21), cardiac death (RR: 0.67, 95% CI: 0.33–1.39; *P* = 0.28), myocardial infarction (RR: 0.70, 95% CI: 0.35–1.42; *P* = 0.33), stroke (RR: 0.75, 95% CI: 0.44–1.26; *P* = 0.28), all bleeding events (RR: 1.11, 95% CI: 0.81–1.53; *P* = 0.51) and major bleeding events (RR: 0.91, 95% CI: 0.56–1.49; *P* = 0.71) were not significantly different as shown in Fig. 3.

The main results of this analysis have been tabulated (Table 5).

**Table 5:** Main results of this analysis

Endpoints assessed	RR with 95% CI	P value	I^2^ value (%)
**Composite endpoints**	0.82 [0.70–0.96]	0.01	34
**Heart failure**	0.66 [0.47–0.92]	0.02	0
**Atrial fibrillation**	0.77 [0.63–0.94]	0.01	33
**Re-admission**	0.78 [0.62–0.99]	0.04	70
**All-cause mortality**	0.86 [0.68–1.09]	0.21	0
**Cardiovascular death**	0.67 [0.33–1.39]	0.28	65
**Myocardial infarction**	0.70 [0.35–1.42]	0.33	8
**Stroke**	0.75 [0.44–1.26]	0.28	21
**Any bleeding event**	1.11 [0.81–1.53]	0.51	0
**Major bleeding events**	0.91 [0.56–1.49]	0.71	0

*R*R Risk ratios, *CI* Confidence intervals

Sensitivity analysis was carried out and it resulted in consistent results throughout the outcome related subgroup analyses. The results which were obtained were not significantly different from the main results of this analysis.

**Fig. 4:**
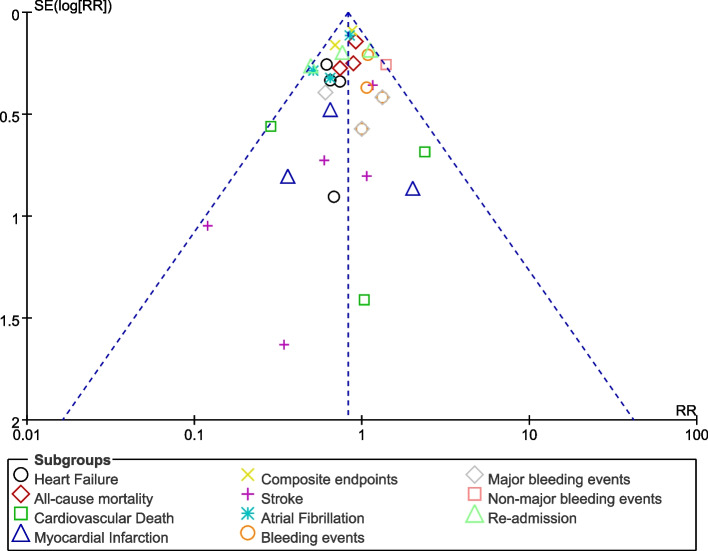
Funnel plot representing publication bias

A visual estimation of the funnel plot also showed low evidence of publication bias as shown in Fig. 4.

## Discussion

In this analysis, we aimed to show the impact of a nurse-led interventional program in improving clinical outcomes in the general population with AF. Our results showed that the risks of heart failure, recurrent AF and re-admission were significantly reduced in the nurse-led interventional group when compared to the usual care group.

Several studies have shown the importance of a nurse-led interventional program in patients with AF. A study published in the year 2020 showed that nurses could help improve self-care of patients living with AF [20]. Even though most participants had adequate health literacy, most reported gaps in AF knowledge. Moreover, Yeager et al. showed that a nurse-led risk factor modification program could well improve weight loss and obstructive sleep apnea in patients with AF thus improving the quality of life of such patients [21]. The study consisted of 189 patients with obesity and 93 patients with obstructive sleep apnea enrolled during a 1-year period between November 2016 up to October 2017.

A systematic-mixed studies review which aimed to provide the first synthesis of evidence for the impact of nurse-led atrial fibrillation clinics on patients, healthcare utilization and quality of care outcomes showed nurse-led clinics to improve healthcare and patients outcomes [22]. The authors stated that nurse-led atrial fibrillation clinics proved to be more cost-effective, reduced waiting time, decreased hospitalization and emergency visits. In addition, another study which was a cost-effective analysis alongside a randomized trial with 712 participants at the Maastricht University Medical Center whereby patients were allocated to a nurse-led care versus a usual care, the nurse-led care was associated with slightly more life years and quality adjusted life years at a lower cost showing a nurse-led care to be dominant, and thus considered to save cost and improve survival as well as quality of life [23]. In the United States, the implementation of a nurse-led AF clinic could reduce hospitalization and emergency department visits as well [22].

In addition, a systematic review and meta-analysis based on a multi-disciplinary integrated care in AF also supported the results of this current analysis showing a decrease in cardiovascular hospitalization [6]. However, the multi-disciplinary integrated team consisted of general practitioners, dietitians, pharmacists, social workers and nurses working together as a team whereas our current analysis strictly focused on nurse intervention without the involvement of other medical staffs.

The Nurse-led multi-component behavioral activation (N-MBA) program based on knowledge of AF, health-related quality of life, and medication adherence in patients with AF represented a feasible and acceptable method which could significantly improve the quality of life of patients with AF [24]. Patients received regular telephone calls by nursing staffs and we regularly reminded about self-care and were also regularly educated about their disease. This practice was well accepted and improved quality of life of patients with AF. Also, in the ALL-IN cluster trial which was based on an integrated management of AF in the primary care, the authors showed that there was a reduction in 45% of all-cause mortality among the elder patients with AF who were assigned to the integrated care setting when compared to the usual care setting [25]. However, the study was monitored by general practitioners as well as nurses working together whereas our current study focused only on nurse intervention.

The SPOT-AF prospective study demonstrated a nurse-led smartphone electrographic monitoring for AF after ischemic stroke [26]. The authors showed that among 1079 participants who were monitored with this electrographic technique, AF could easily be detected in comparison to the 24 hours Holter monitoring. This method, monitored by nurses, could better detect recurrent AF when compared to the Holter monitoring device.

At last, even though a nurse-led interventional program or a nurse-led clinic could significantly improve clinical outcomes and decrease re-admission of patients with AF, this nurse-led program could be more applicable to low-risk patients with AF [27]. Or else, this could lead to a delay in appropriate assessment and management of high-risk AF patients. In addition, there is still a need to improve cardiovascular nurses’ knowledge and practices related to AF, stroke prevention and anticoagulation therapy [28]. Also, a clinical supervision model on nurse performance in the care of patients with AF could be considered to provide effective staffs [29]. Education courses for nurses could also prove effective in improving nurses’ knowledge on AF and its treatment [30].

### Limitations

This study also has limitations. The total number of participants were limited to 2916 and this low number of participants could impact the final results. Moreover, several endpoints were not reported in all the studies. For example, study A reported outcomes X, Y and Z whereas study B only reported outcome X, without outcomes Y and Z. Therefore, an analysis including endpoint Y and Z consisted of less studies and therefore this could also have an impact on the final results. In addition, one study was an observational study and data from observational study could lead to the introduction of selection, and other bias. However, this observational study did not have any major influence on the results since its data did not impact our sensitivity analysis. Also, the follow-up time period, the cardiac medications and anticoagulants which were used were ignored when carrying out this analysis. Another limitation could be the fact that patients with new onset AF, chronic AF, paroxysmal AF, non-valvular and valvular AF were combined and analyzed. This was due to a lack of studies based on different types of AF patients.

## Conclusions

The nurse-led interventional program significantly improved composite endpoints including heart failure and the recurrence of AF, resulting in a significantly lower admission rate compared to the usual care group. However, nurse-led interventional program did not affect mortality, stroke, myocardial infarction and bleeding events. Based on our current results, a nurse-led interventional programs apparently could be beneficial in patients with AF. Future larger trials would be able to confirm this hypothesis.

## Data Availability

Data which have been used in this study can freely be accessed and are included in the original published articles. References of the original papers involving the data source which have been used in this paper have been listed in the main text of this current manuscript. All data are publicly available in electronic databases.
